# CD3+CD4+LAP+Foxp3-Regulatory Cells of the Colonic Lamina Propria Limit Disease Extension in Ulcerative Colitis

**DOI:** 10.3389/fimmu.2018.02511

**Published:** 2018-10-30

**Authors:** Alessia Butera, Massimo Sanchez, Annamaria Pronio, Antonello Amendola, Daniela De Nitto, Nazzareno Di Carlo, Roberto Lande, Loredana Frasca, Francesco Borrini, Roberta Pica, Monica Boirivant

**Affiliations:** ^1^Pharmacological Research and Experimental Therapy Section, National Center for Drug Research and Evaluation, Istituto Superiore di Sanità, Rome, Italy; ^2^Cytometry Unit-Core Facilities, Istituto Superiore di Sanità, Rome, Italy; ^3^Department of General Surgery “P. Stefanini”, Sapienza University, Rome, Italy; ^4^Unit of Arbo, Hanta and Emerging Viruses, National Reference Laboratory for Arboviruses, Department of Infectious Diseases, Istituto Superiore di Sanità, Rome, Italy; ^5^IBD, GE Unit, Sandro Pertini Hospital, Rome, Italy; ^6^UOC Pathology Section, Sandro Pertini Hospital, Rome, Italy

**Keywords:** CD3+CD4+LAP+Foxp3-regulatory cells, inflammation, immunity, oxazolone colitis, regulatory T cells, ulcerative colitis

## Abstract

**Background and Aims:** In ulcerative colitis (UC), inflammation begins in the rectum and can extend proximally throughout the entire colon. The extension of inflammation is an important determinant of disease course, and may be limited by the action of regulatory T cells (Tregs). In this cross-sectional study, we evaluated the relationship between UC extension and the proportions of CD3+CD4+Foxp3+ and CD3+CD4+LAP+Foxp3-Tregs in the colonic lamina propria (LP) of 79 UC patients and 29 controls. The role of these cells in UC extension was also investigated in the murine oxazolone-induced colitis model.

**Methods:** Patients: Disease extension was classified according to the Montreal classification. Where possible, endoscopic biopsies of involved and uninvolved tissue were obtained from UC patients. Mouse model: Colitis was induced by intrarectal oxazolone administration. Lamina propria mononuclear cells were isolated from patient biopsies and mouse colon tissue using enzymatic method and the percentage of CD3+CD4+Foxp3+ and CD3+CD4+LAP+Foxp3-cells evaluated by immunofluorescence. Confocal microscopy was applied for the visualization and quantification of CD4+LAP+ cells on tissue histological sections.

**Results:** In UC patients with distal colitis the proportion of LP CD3+CD4+Foxp3+ Tregs was significantly higher in inflamed tissue than uninvolved tissue. As opposite, the proportion of LP CD3+CD4+LAP+ Tregs was significantly higher in uninvolved tissue than involved tissue. Both LP CD3+CD4+Foxp3+ and LP CD3+CD4+LAP+ Tregs proportion in involved tissue was significantly higher than in controls irrespective of the extension of inflammation. In mice with oxazolone-induced distal colitis, treatment with LAP-depleting antibody was associated with the development of extensive colitis.

**Conclusions:** Our findings suggest that CD3+CD4+LAP+Foxp3-Tregs limit the extension of inflammatory lesions in UC patients.

## Introduction

Ulcerative colitis (UC) is characterized by inflammation, typically restricted to the mucosal surface that begins in the rectum and can continuously extend proximally throughout the entire colon (1). A prospective study of Norwegian UC patients found that at the time of presentation, colitis was limited to the rectum (proctitis) in one third of patients, the colorectum distal to the splenic flexure (left-sided colitis) in another third, and the area proximal to the splenic flexure (extensive colitis) in the remaining third (2). In patients with distal colitis, inflammation progresses in 25–50% of patients, while regression is observed in about 16% of patients (3, 4). The anatomical extent of mucosal inflammation is one of the most important factors determining disease course. Patients with extensive colitis have a greater risk of colectomy than those with proctitis (5–7), as well as a greater risk of colorectal cancer (8, 9). Proximal disease extension is associated with increased disease severity upon diagnosis and greater likelihood of clinical relapse (10, 11). Moreover, extensive colitis is associated with an increased frequency of extraintestinal manifestations, a steroid-refractory disease course, and the need for immunosuppressive and immune-modulating medications and surgery (10, 12). Younger age at diagnosis and the presence of sclerosing cholangitis are pre-existing independent predictive factors for disease progression (12).

The biological factors that determine the extent of inflammation in UC are unknown. Tregs have been identified as a key immunosuppressive population that is critically involved in maintaining intestinal homeostasis. Therefore, we hypothesized that Tregs may be involved in limiting the extension of inflammation. To investigate this hypothesis, we evaluated the frequencies of different Treg types in the lamina propria (LP) of UC patients with varying degrees of disease extension, and examined the contribution of these cells to disease extension in a mouse model of UC. Specifically, we analyzed the frequencies of CD3+CD4+Foxp3+ cells, as well as another type of Treg, CD3+CD4+ latency associated peptide (LAP)+ Foxp3-regulatory cells. We previously demonstrated that the latter cell type is present in human LP (13), and is found at a higher frequency in the LP of endoscopically active UC patients, but not in Crohn's disease patients (14). Moreover, LAP^+^ Foxp3^−^ T cells have been described to infiltrate colorectal cancer exhibiting more potent immunosuppressive activity than Foxp3^+^ regulatory T cells (15).

We found that in UC patients with proctitis and left-sided colitis the proportion of LP CD3+CD4+Foxp3+ Tregs was significantly higher in inflamed tissue than uninvolved tissue. As opposite, the proportion of LP CD3+CD4+LAP+ Tregs was significantly higher in uninvolved tissue than involved tissue. In a mouse model of distal colitis (16), we found that administration of an LAP-depleting antibody that has no effect on the frequency of CD4+Foxp3+ Tregs (17) was associated with the development of extensive colitis, suggesting that CD3+CD4+LAP+Foxp3-regulatory cells limit the extension of inflammatory lesions in UC.

## Materials and methods

### Patients

A total of 95 patients with endoscopically active UC who underwent colonoscopy for clinical flare-up at 2 tertiary centers (IBD, GE Unit, Sandro Pertini Hospital, Rome, and the Department of General Surgery, “P. Stefanini,” Sapienza University, Rome) were included in the study. Disease activity was assessed using the endoscopic Mayo score (18). Patients with an endoscopic score ≥1, not on rectal 5-ASA and/or steroids in the last 3 months, were considered to have endoscopically active UC and were enrolled in the study. The control group consisted of 29 participants [14 men, 15 women; mean ± SE age, 55 ± 3 years; median (range) age, 56 (26–85)] undergoing colonoscopy for colonic cancer screening and suspected functional bowel disorders. In UC patients, disease extension, at time of endoscopy, was classified using the Montreal classification (19) as follows: proctitis, involvement limited to the rectum; left-sided colitis, involvement limited to a portion of the colorectum distal to the splenic flexure; extensive colitis, involvement extending proximal to the splenic flexure.

### Biopsy specimens

Multiple endoscopic mucosal biopsies were obtained. Biopsies were taken from endoscopically involved and uninvolved areas in UC patients, and from matched areas in controls. Diagnosis of UC was established based on established criteria and the localization and extension of the disease were confirmed by histology. Histopathology was quantified in H&E stained tissue sections using the Robarts histopathology index (RHI) (20). An RHI ≤ 6 in samples collected from involved tissue was considered indicative of remission, and the sample, together with the paired sample collected from uninvolved tissue, was excluded from the analysis. Accordingly, 16 patients with a Mayo endoscopic score = 1 were excluded from the analysis. The remaining 79 patients were evaluated applying the aforementioned criteria. In these patients, RHI < 3 were recorded for all biopsy samples collected from uninvolved tissue. Histology confirmed the absence of inflammatory changes in controls.

Some tissue sections were also analyzed by confocal microscopy. Three micrometer thick paraffin-embedded sections of human colon tissue from controls (CTR) and ulcerative colitis (UC) patients were stained after deparaffinization in xylene (5 min, two times), rehydratation by sequential washes in 100% ethanol (3 min), 95% ethanol (3 min), 80% ethanol (3 min), 70% ethanol, 50% ethanol, deionized water and antigen retrieval (5 min at 95°C in 10 mM sodium citrate, pH 6.0). Slides were saturated with blocking buffer (PBS, 0.05% tween 20, 4% BSA) for 1 h at room temperature. Specimens were stained with a polyclonal rabbit anti-human CD4 at 5 μg/ml, followed by donkey anti-rabbit-AlexaFluor-568, and a monoclonal mouse anti-human LAP followed by an goat anti-mouse AlexaFluor-647. After washing, slides were mounted in Prolong Gold anti-fade medium containing a DNA dye (DAPI). Confocal laser scanning microscopy (CLSM) observations were performed with a Leica TCS SP2 AOBS apparatus, using a 63x/1.40 NA oil objective. Acquisition of images was performed by a Leica confocal software 2.6 (Leica, Germany).

Clinicopathological variables for all patients are shown in Table 1.

**Table 1:** Patient clinicopathological variables.

**Disease extension (*n*)**	**Age (years) mean ± SE median (range)**	**Sex M/F (*n*)/(*n*)**	**Disease duration since diagnosis (years) mean ± SE median (range)**	**Mayo endoscopic score mean ± SE median (range)**	**Therapy**			
					**CS**	**5-ASA agents**	**Immuno-modulators**	**Biological agents**
Proctitis (21)	49 ± 3 52 (20–76)	17/14	10 ± 2 7 (0–35)	2 ± 0.12(1–3)	5^*^+1	24	1	1
Left-sided Colitis (22)	53 ± 3 54 (23–82)	21/11	12 ± 2 12 (0–37)	2 ± 0.12(1–3)	4^*^	22	6^*^
Extensive Colitis (16)	43 ± 5 46 (19–70)	9/7	10 ± 2 15 (0–24)	2 ± 0.22(1–3)	2^*^+1	10	1^*^+1	1^*^+1

* *Patients with combined therapy*.

### Isolation of lamina propria mononuclear cells (LPMCs)

LPMCs were isolated from freshly obtained biopsies using a previously described DDT-EDTA collagenase method (13, 14). Briefly, biopsies were washed in HBBS free of calcium and magnesium (HBSS-CMF; Hyclone, Europe LTD, Cramlington, United Kingdom), and then incubated for 5 min at room temperature in HBSS-CMF containing 1 mmol/l DTT (Sigma Chemical Co., St. Louis, MO, United States) and antibiotics (penicillin, 100 U/ml; streptomycin, 100 mg/ml; gentamicin, 50 mg/ml; and fungizone, 25 mg/ml). After washing 3 times in HBSS-CMF, the biopsies were cut into smaller pieces and incubated in HBSS-CMF containing 0.75 mmol/l EDTA, 10 mmol/l HEPES buffer, and antibiotics for 15 min at 37°C in a humid 5% CO_2_ atmosphere to remove epithelial cells. After 2 washes, the tissue was incubated for a total of 2 h (2 × 1-h incubations) at 37°C in a humid 5% CO_2_ atmosphere in complete medium (RPMI 1640 plus 10 mM HEPES buffer, 2 mM l-glutamine, 10% heat-inactivated FCS (Hyclone), and antibiotics) containing 25 U/ml collagenase V (Sigma-Aldrich, Milan, Italy) and 100 μg/ml of DNase (Roche Diagnostics, Mannheim, Germany). After incubation, the supernatant containing LMPCs was collected and washed twice in HBSS-CMF + antibiotics.

### Antibodies and reagents

APC-Cy7-labeled anti-CD3, PE-Cy7-labeled anti-IL-10, and isotype-matched control Igs were obtained from Biolegend (San Diego, CA, United States). PE-labeled anti IL-17A, and isotype-matched Ig control were obtained from eBioscience [San Diego, CA, United States]. FITC-labeled anti-CD4, PE-CF594-labeled anti-CD8 and isotype-matched control Ig were obtained from Becton Dickinson Horizon (San Jose, CA, United States). PerCP-labeled anti-LAP [TGF-β1] and isotype-matched control Ig were obtained from R&D Systems (Minneapolis, MN, United States). APC-labeled anti-Foxp3, the Foxp3 staining buffer set, and isotype-matched control Ig were obtained from eBioscience (San Diego, CA, United States). LIVE/DEAD ® Fixable Aqua Dead Cell Stain Kit was obtained from Life Technologies (Carlsbad, CA, United States). Phorbol-12-myristateacetate (PMA) and ionomycin were obtained from Sigma-Aldrich. FITC-labeled anti-CD4, isotype-matched control Igs, and Monensin solution (Golgi Stop) were obtained from BD Pharmingen (San Jose, CA, United States). For confocal microscopy imaging, polyclonal rabbit anti-CD4 (Novus, Colorado, United States), Donkey anti-rabbit-AlexaFluor-568 (Abcam, Cambridge, United Kingdom), monoclonal mouse anti-human LAP (R&D Systems) and goat anti-mouse AlexaFluor-647 (Abcam) were used.

### Immunofluorescence staining

Isolated LPMCs were incubated for 30 min with LIVE/DEAD® Fixable Aqua Dead Cell Stain. Next, cells were washed and stained with anti-human-CD3, anti-human-CD4, and anti-human LAP (TGF-β1). After incubation, cells were washed, fixed, and permeabilized with fixation/permeabilization solution for 40 min, and stained with anti-human Foxp3. In previous studies (14), we established that the percentages of LAP+ and Foxp3+ cells remained unchanged following PMA-ionomycin stimulation in the presence of Golgi Stop. Therefore, for evaluation of LAP and Foxp3 expression, together with that of the intracellular cytokine IL-10 and IL-17, LPMCs isolated from biopsies were incubated in X-VIVO15 medium and stimulated for 4 h with PMA (50 ng/ml) and ionomycin (1 μg/ml) in the presence of monensin (0.66 μl/ml Golgi Stop). After stimulation, cells were recovered and washed in PBS-1X, incubated for 30 min with LIVE/DEAD® Fixable Aqua Dead Cell Stain, and washed and labeled. Given the downregulation of CD4 expression that occurs following PMA-ionomycin stimulation, CD8 staining was preferred. Consequently, to assess intracellular cytokine expression, cells were stained with anti-human CD3, anti-human CD8, and anti-human LAP (TGF-β1), and incubated for 30 min. After incubation, cells were washed, fixed, and permeabilized with fixation/permeabilization solution for 40 min. Cells were then washed in permeabilization buffer and incubated for 30 min with intracellular anti-human IL-10, anti-human IL-17 and anti-human Foxp3 or isotype-matched control Ig. Next, cells were washed twice with permeabilization buffer solution and fixed with 2% paraformaldehyde. CD4 cells were defined as those with a CD3+CD8-signature. The percentage of viable fluorescent cells was quantified using a Gallios™ Flow Cytometer (Beckman Coulter, Brea, CA, United States).

### Induction of experimental colitis in mice

Male BALB/c mice (Charles River Laboratories Italia, Calco, Italy) were housed in the Allevamenti Plaisant (Rome, Italy) animal facility in individually ventilated cages (IVC system) containing enrichment devices. Maintenance of pathogen-free conditions was ensured by monitoring every 6 months, in accordance with the Federation of European Laboratory Animal Science Associations (FELASA) recommendations. Experimental colitis was induced in 6–7 week-old male BALB/c mice by administering 6 mg of oxazolone [(4-ethoxymethylene-2-phenyl-2-oxazolin-5-one) Sigma Chemical Co., St. Louis, MO, United States] dissolved in 50% ethanol (total injection volume,150 μl), via a 3.5-F catheter inserted into the rectum of lightly anesthetized mice, as previously described (15). Control groups consisted of untreated mice and mice treated with 50% ethanol (total injection volume, 150 μl). Body weight was recorded at time zero (moment of intrarectal oxazolone/ethanol or ethanol administration), and on days 1 and 2 post-treatment. Mice were sacrificed on day 2 by cervical dislocation and colons collected for further analysis. In a preliminary experiment, we tested the ability of anti-LAP antibody (TW7-16B4 antibody, kindly donated by Professor HL Weiner, Harvard Medical School, Boston, MA, USA) to deplete the LP CD4+LAP+cells. To this end we treated two different groups of mice for 5 days with daily injection (40 μg, i.p.) of anti-LAP antibody or isotype control mouse IgG1 Clone MOPC-21 (BioXCell; DBA, Segrate, MI). Mice were sacrificed, colons were collected, and isolated LPMC cells were analyzed by immunofluorescence for LAP and Foxp3 expression. In additional experiments, different groups of mice received daily injections (40 μg, i.p.) of anti-LAP antibody (or isotype control), for 5 days before the induction of colitis.

### Assessment of colitis

Collected colons were macroscopically examined to assess the extension of colitis, and histological analysis of colitis was performed in colonic tissue samples that were fixed in 10% neutral buffered formalin solution (Sigma-Aldrich), embedded in paraffin, cut into tissue sections, and stained with hematoxylin and eosin. Multiple serial sections dividing the colon into three sections (proximal, medial and distal) were performed. Stained sections were examined by a pathologist [ISTOVET di Luca Crippa and C. S.A.S., Besana in Brianza (MB), Italy] and the extension of inflammatory lesions determined. Histopathologic grading of oxazolone-induced colitis was performed as previously described (23). Briefly, 5 criteria (hypervascularization, presence of mononuclear cells, epithelial hyperplasia, epithelial damage, presence of granulocytes and mucosal hemorrhages) were scored from 0 to 3 to produce a cumulative histopathologic score (HS) ranging from 0 (no colitis) to 15 (maximal colitis activity).

### LPMCs immunofluorescence staining

Freshly isolated and washed LPMCs were subjected to Fc block with anti-CD16/CD32 (BD Pharmingen) and then labeled for 30 min with LIVE/DEAD® Fixable Aqua Dead Cell Stain. After washing, cells were stained by incubation for 30 min with APC-Cy7-labeled anti-CD3 (eBioscience), V450-labeled anti-CD4 (eBioscience), PE-labeled anti-LAP (BioLegend), or isotype control PE-labeled mouse IgG1 (R&D Systems). Intracellular Foxp3 expression was evaluated using the APC-anti-mouse/rat Foxp3 staining kit (eBioscience), following the manufacturer's protocol. The cells were then washed twice, and the percentage of fluorescent cells quantified using a Gallios™ Flow Cytometer (Beckman Coulter, Brea, CA, United States).

Some tissue sections were also analyzed by confocal microscopy, using the above-described procedure. Briefly, 3 μm-thick paraffin-embedded sections of unlesional and lesional colon tissues from mice treated as described in Induction of experimental colitis in mice paragraph were stained after deparaffinization, rehydratation, antigen retrieval and saturation with blocking buffer. Specimens were stained with a monoclonal FITC-conjugated rat anti-mouse CD4 at 5 μg/ml (BD Biosciences), and a monoclonal mouse anti-mouse LAP at 5 μg/ml (BioLegend) followed by an goat anti-mouse AlexaFluor-633. After washing, slides were mounted in Prolong Gold anti-fade medium containing a DNA dye (DAPI). Confocal laser scanning microscopy (CLSM) observations were performed with a Leica TCS SP2 AOBS apparatus, using a 63x/1.40 NA oil objective. Acquisition of images was performed by a Leica confocal software 2.6 (Leica, Germany).

### Statistical analysis

Data were analyzed using the two sided Mann-Whitney *U*-test and the two sided Wilcoxon signed-rank test in GraphPad Prism software (GraphPad Software, San Diego, CA, United States). Statistical significance was set at *p* < 0.05.

### Human study

All participants provided written informed consent prior to inclusion in the study. Ethical approval was provided by the Ethical Committee of the Istituto Superiore di Sanità (Reference: Pre-C-871/14, 25/11/2014).

### Animal studies

This study was carried out in accordance with the recommendations of Decreto Legislativo 4 marzo 2014, n. 26 according with 2010/63/UE(14G00036) direction. The protocol was approved by the Italian Ministry of Health (Reference: 16/2014-PR [DGSAF 12073-A, 05/06/2014], 03/10/2014).

## Results

### The frequency of LP CD3+CD4+LAP+ cells is higher in uninvolved vs. involved colon tissue from ulcerative colitis patients

**Figure 1:**
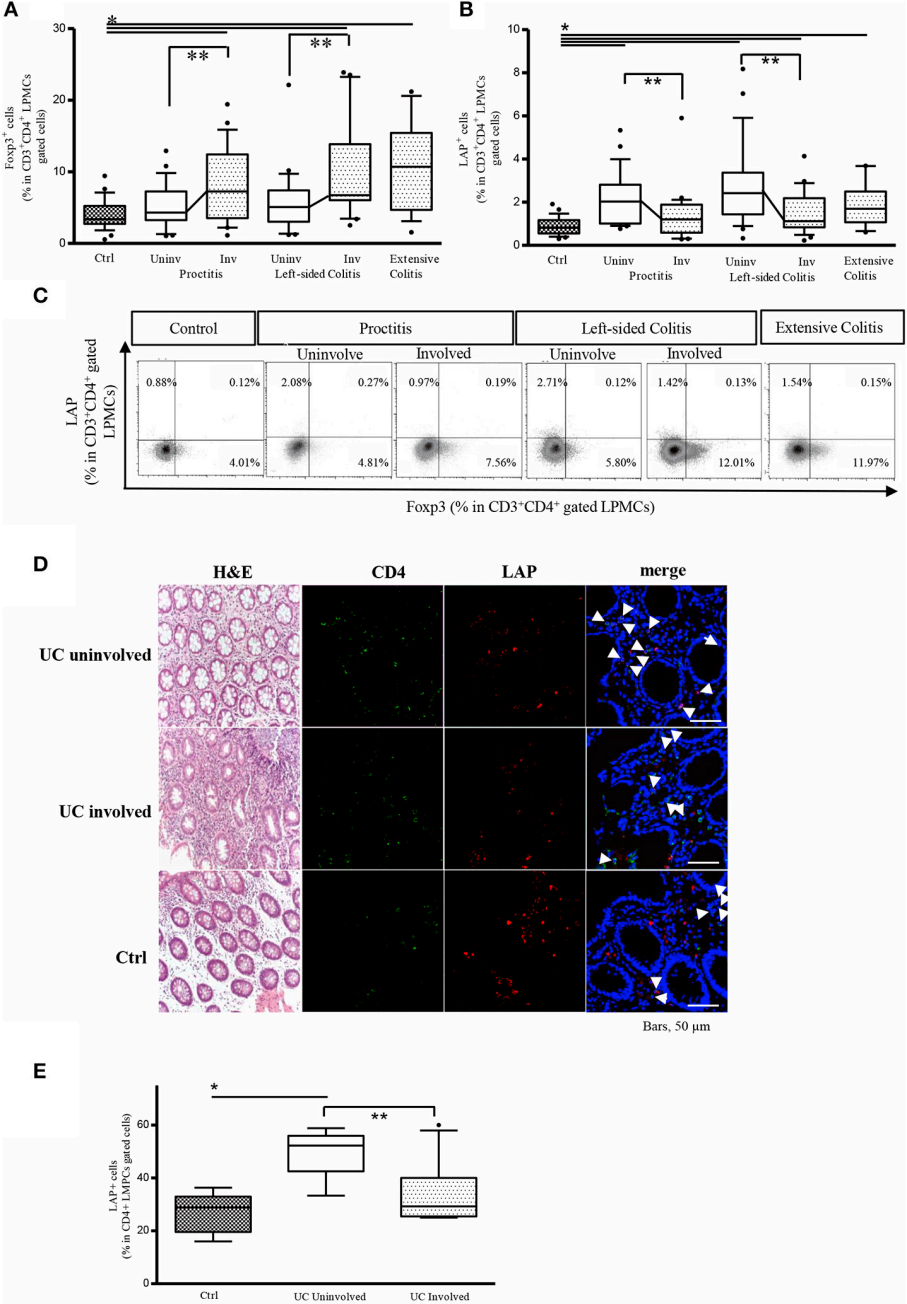
The frequency of LP CD3+CD4+LAP+ cells is higher in uninvolved (Uninv) vs. involved (Inv) colon tissue from UC patients. **(A)** Frequency of LP Foxp3+ cells in the CD3+CD4+-gated LPMC population: *P < 0.05 (Mann-Whitney *U*-test) for controls (Ctrl) vs. proctitis (Uninv + Inv), left-sided colitis (Inv), and extensive colitis; **P<*0.05* (Wilcoxon signed-rank test) for uninvolved vs. involved tissue in proctitis and left-sided colitis. Data represent the mean ± SE of 79 UC patients and 29 controls. **(B)** Frequency of LP LAP+ cells in the CD3+CD4+-gated LPMC population: *P<*0.05* for Ctrl vs. proctitis (Uninv + Inv) and left-sided colitis (Inv); *P<*0.05* (Mann-Whitney *U*-test) for controls vs. left-sided colitis (Uninv + Inv) and extensive colitis; **P<*0.05* (Wilcoxon signed-rank test) for uninvolved vs. involved tissue in proctitis and left-sided colitis. Data represent the mean ± SE of 79 UC patients and 29 controls. **(C)** Representative density plots of Foxp3 and LAP expression in LP CD3+CD4+-gated cells. **(D)** Representative CD4+LAP+ T cells in colonic mucosa tissue of UC patients and controls. Confocal microscopy images of CD4 (green), LAP (red) and nuclei (blue) of matched involved and uninvolved colonic mucosa of a patient with UC, and a control subject (original magnification 630x). CD4+LAP+ double positive T cells are indicated by white arrows. For UC, 1 representative staining of 4 patients is shown. For control subjects, 1 representative staining of 2 subjects is shown. H&E stained sections of the corresponding subjects are also illustrated (original magnification 200x). **(E)** % of CD4+LAP+ cells in UC patients (involved and uninvolved tissue) and controls. For quantification at least 3 images for each patient (*n* = 4) or control subjects (*n* = 2) were assessed. Data represent mean ± SE, *P<*0.05* (Mann-Whitney *U*-test) for controls vs. uninvolved tissue; **P < 0.05 (Wilcoxon signed-rank test) for uninvolved vs. involved tissue.

Preliminary analyses of the percentage of CD3+CD4+LAP+ and CD3+CD4+Foxp3+ Tregs isolated from biopsies from different portions of control colons revealed no differences between colon regions (Supplementary Figure 1). We next evaluated the frequency of LP CD3+CD4+LAP+ and CD3+CD4+Foxp3+ Tregs in LPMCs isolated from biopsies from controls and from patients with endoscopically and histologically active UC with varying degrees of disease extension. In patients with either proctitis or left-sided colitis, the percentage of CD4+Foxp3+ cells was significantly higher in involved tissue than in uninvolved tissue (Figure 1A). As opposite % of CD4+LAP+ T regs was significantly higher in uninvolved vs. involved tissue (Figure 1B) In agreement with previous observations (14, 24, 25), the percentage of Foxp3+ Tregs in the LP CD3+CD4+ T-cell population was significantly higher in involved tissue, regardless of disease extension, than in controls (Figure 1A). Similarly, in line with previous reports (14), the percentage of CD4+LAP+ Tregs was significantly higher in involved tissue from patients with extensive colitis and left-sided colitis than in controls (Figure 1B). In uninvolved tissue, the percentage of CD4+Foxp3+ cells was comparable to that of controls, while the percentage of CD4+LAP+ Tregs tissue was significantly higher. As previously reported (14), the majority of LAP+ cells detected were Foxp3- (Figure 1C). Some biopsy specimens sections were also immunofluorescence stained for tissue assessment of CD4+LAP+ cells by confocal microscopy. As illustrated in Figures 1D,E, uninvolved tissue showed significantly more CD4+LAP+ double-fluorescent cells when compared with involved and control tissue.

### Ratio between IL-10 and IL-17 expressing LAP+ cells is significantly higher in uninvolved vs. involved colon tissue from ulcerative colitis patients

**Figure 2:**
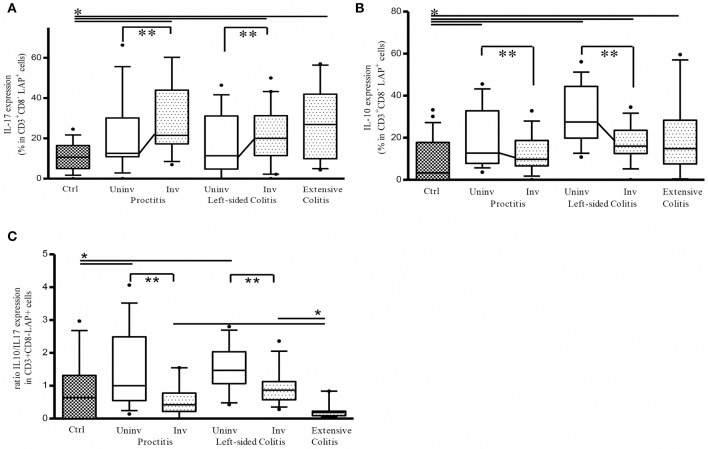
Ratio between IL-10 and IL-17 expressing CD3+CD8-(CD4)LAP+ cells is significantly higher in uninvolved (Uninv) vs. involved (Inv) colon tissue from ulcerative colitis patients. **(A)** Frequency of LP LAP+ cells expressing intracellular IL-17 *P < 0.05 (Mann-Whitney *U*-test) for controls vs. Involved (Inv) tissue in proctitis, left sided colitis and extensive colitis. **P < 0.05 (Wilcoxon signed-rank test) for uninvolved vs. involved tissue in both proctitis and left-sided colitis. **(B)** Frequency of LP LAP+ cells expressing intracellular IL-10: *P < 0.05 (Mann-Whitney *U*-test) for controls vs. proctitis (Uninv), left-sided colitis (Uninv + Inv), and extensive colitis; **P < 0.05 (Wilcoxon signed-rank test) for uninvolved vs. involved tissue in both proctitis and left-sided colitis. **(C)** % LP LAP+ cells expressing intracellular IL-10 / % LP LAP+ cells expressing intracellular IL-17 (ratio). **P < 0.05 (Wilcoxon signed-rank test) for uninvolved vs. involved tissue in proctitis, and left sided colitis. *P < 0.05 (Mann-Whitney *U*-test) proctitis and left-sided colitis univolved tissue vs. controls *P < 0.05 (Mann-Whitney *U*-test) Extensive colitis vs. proctitis and vs. left-sided colitis involved tissue. Data represent mean ± SE of 43 UC patients (proctitis, 16; left-sided colitis, 17; extensive colitis, 10) and 26 controls.

We have previously shown that in active UC patients, LP CD3+CD8- (CD4) LAP+ cells are enriched for IL-17 expression, showing reduced suppressor activity due the intracellular IL-17 expression (14). It has been recently reported that CD4+LAP+ cells expressing IL-10 exhibit regulatory activity (26, 27). We therefore evaluated IL-17 and IL-10 expression in LP CD3+CD8- (CD4) LAP+ cells. As shown in Figure 2A, the percentage of IL-17 expressing LP CD3+CD8- (CD4) LAP+ cells was significantly reduced in uninvolved vs. involved tissue while the % of IL-10-expressing LAP+ cells was significantly higher in uninvolved vs. involved tissue (Figure 2B). As a consequence, the ratio between IL-10 and IL-17 expressing LAP+ cells was significantly higher in uninvolved vs. involved tissue (Figure 2C). Notably, extensive colitis ratio was significantly lower than the ratio observed both in proctitis and left-sided colitis involved tissue. As previously reported and confirmed in the present study, % of IL-17 expressing LAP+ cells was significantly increased in involved tissues vs. controls (14). % of IL-10 expressing LAP+ cells was significantly increased in uninvolved tissues vs. controls.

### Reduced disease extension is associated with a higher percentage of CD3+CD8– (CD4+) LAP+ cells in uninvolved vs. involved tissue from ulcerative colitis patients

**Figure 3:**
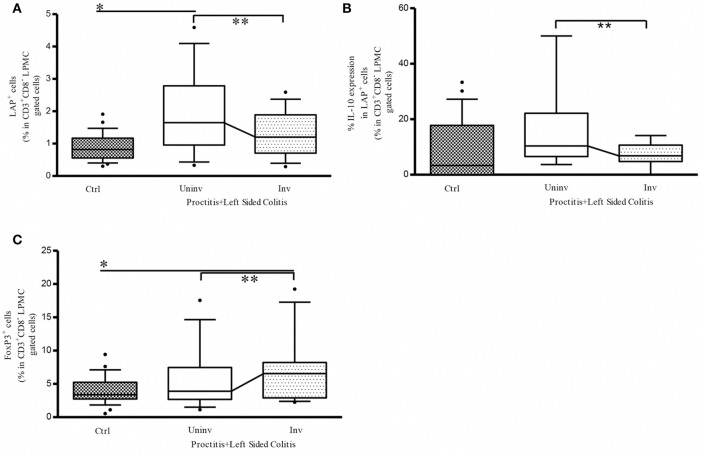
Reduced disease extension is associated with a higher percentage of CD3+CD8-(CD4) LAP+ cells in uninvolved vs. involved colon tissue from UC patients. **(A)** Frequency of LP LAP+ cells in CD3+CD8- (CD4)-gated cells: *P < 0.05 (Mann-Whitney *U*-test) for controls vs. uninvolved tissue in proctitis + left-sided colitis; **P < 0.05 (Wilcoxon signed-rank test) for uninvolved vs. involved tissue in proctitis + left-sided colitis. **(B)** Frequency of LP LAP+ cells expressing intracellular IL-10: **P < 0.05 (Wilcoxon signed-rank test) for uninvolved vs. involved tissue in proctitis + left-sided colitis. **(C)** Frequency of LP Foxp3+ cells in CD3+CD8- (CD4+)-gated cells: **P < 0.05 (Wilcoxon signed-rank test) for uninvolved vs. involved tissue in proctitis + left-sided colitis. In all cases, data represent the mean ± SE of 26 controls and of 13 UC patients with a history of extensive colitis in whom inflammatory lesions were limited to the rectum or left colon at the moment of endoscopy. Patient clinicopathological variables of this UC subgroup are not different from the whole UC patients population [Age: 53 ± 3–53 (29–73) (years) mean ± SE-median (range); Sex: 7/6 M/F; Disease duration since diagnosis:14 ± 3.2–12 (1–32); (years) mean ± SE-median (range). Mayo endoscopic score: 2 ± 0.2–2 (1–3) mean ± SE-median (range)].

To determine whether the observed differences in Tregs frequencies were indeed linked to the extension of inflammatory lesions, we analyzed a subgroup of patients with a history of endoscopic assessed extensive colitis in whom inflammatory lesions were endoscopically limited to the distal colon (mainly rectum) at the moment of the entry in the present study. We found that the presence of distal inflammatory lesions was associated with a significantly higher frequency of LAP+ cells in LP cells isolated from uninvolved tissue vs. both control tissue and involved tissue from UC patients (Figure 3A). Within this cell population, the percentage of IL-10-expressing CD4+LAP+ cells was significantly higher in uninvolved vs. involved tissue (Figure 3B). The percentage of CD4+Foxp3+ Tregs was significantly higher in involved vs. uninvolved tissue, in which values were comparable to those detected in controls (Figure 3C). These findings suggest that the differences in the frequencies of regulatory cells between involved and uninvolved tissue are linked to the extension of inflammatory lesions.

### Depletion of LAP+ cells is associated with proximal extension of inflammatory lesions in the murine oxazolone-induced colitis model

**Figure 4:**
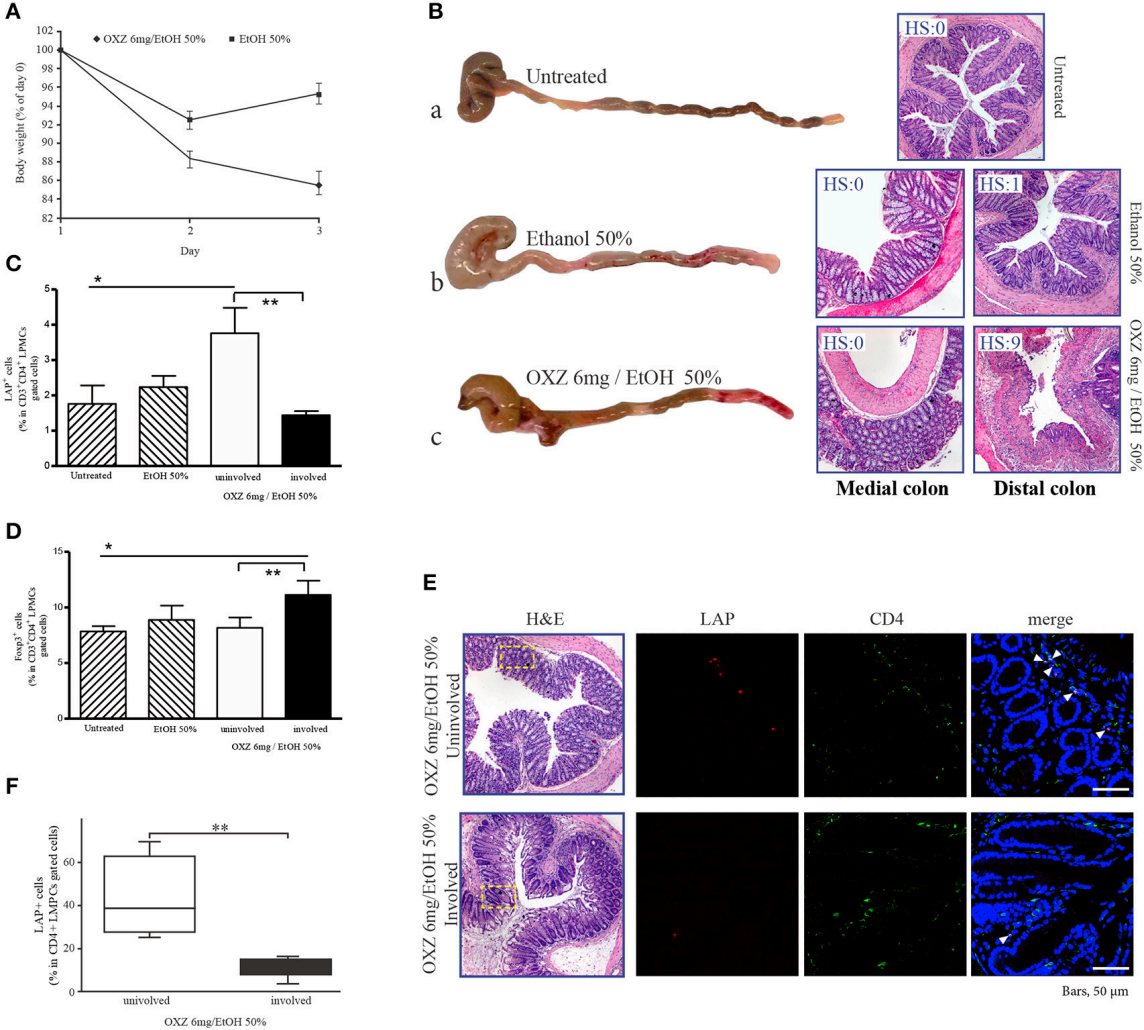
Oxazolone-induced colitis reproduces the observations made in UC patients. **(A)** Weight changes in mice after intrarectal administration of oxazolone (6 mg/ethanol 50%). Each point represents the cumulative mean (± SE) weight from 3 separate experiments in which 5 mice per group were studied. *P < 0.05 (Student's *t*-test) for EtOH 50% vs. OXZ 6 mg/EtOH 50%. **(B)** Representative macroscopic (on the left) and corresponding microscopic (on the right) images of the colons from untreated (a), EtOH 50%-treated (b), and OXZ 6 mg /EtOH 50%-treated (c) mice, all of which were sacrificed 2 days post-treatment. H&E staining of distal (untreated), distal and medial colonic tract of EtOH 50%-treated and oxazolone-treated miceat 40 × magnification. HS, histopathologic score (see methods). **(C)** Percentage of LAP+ cells among CD3+CD4+-gated LMPCs isolated from the colon of untreated, ethanol-treated and oxazolone/ethanol-treated mice: *P < 0.05 for untreated mice vs. OXZ-treated mice (uninvolved tissue); **P < 0.05 for uninvolved vs. involved tissue in OXZ-treated mice. Each point represents mean ± SE of pooled values derived from 3 experiments in which 5 mice per group were evaluated. **(D)** Percentage of Foxp3+ cells among CD3+CD4+-gated LMPCs isolated from the colon of untreated, ethanol-treated, and oxazolone/ethanol-treated mice: *P < 0.05 (Mann-Whitney *U*-test) for untreated mice vs. OXZ-treated mice (involved tissue); **P < 0.05 (Wilcoxon signed-rank test) for uninvolved vs. involved tissue in OXZ-treated mice. Each point represents the mean ± SE of pooled values derived from 3 experiments in which 5 mice/group were evaluated. **(E)** Representative CD4+LAP+ T cells in colonic mucosa of involved and uninvolved tissue of oxazolone-treated mice. Confocal microscopy images of CD4 (green), LAP (red) and nuclei (blue) of matched involved and uninvolved colonic mucosa of a oxazolone-treated mouse (original magnification 630x). CD4+LAP+ double positive T cells are indicated by white arrows. 1 representative staining of 3 mice is shown. H&E stained sections of the corresponding confocal images are also illustrated (original magnification 40x). The rectangles highlight the area shown in the confocal microscopy images. **(F)** % of CD4+LAP+ cells in colons of oxazolone-treated mice (involved and uninvolved tissue). For quantification at least 3 images for each colon (*n* = 3) were assessed. Data represent mean ± SE, **P < 0.05 (Wilcoxon signed-rank test) for uninvolved vs. involved tissue.

**Figure 5:**
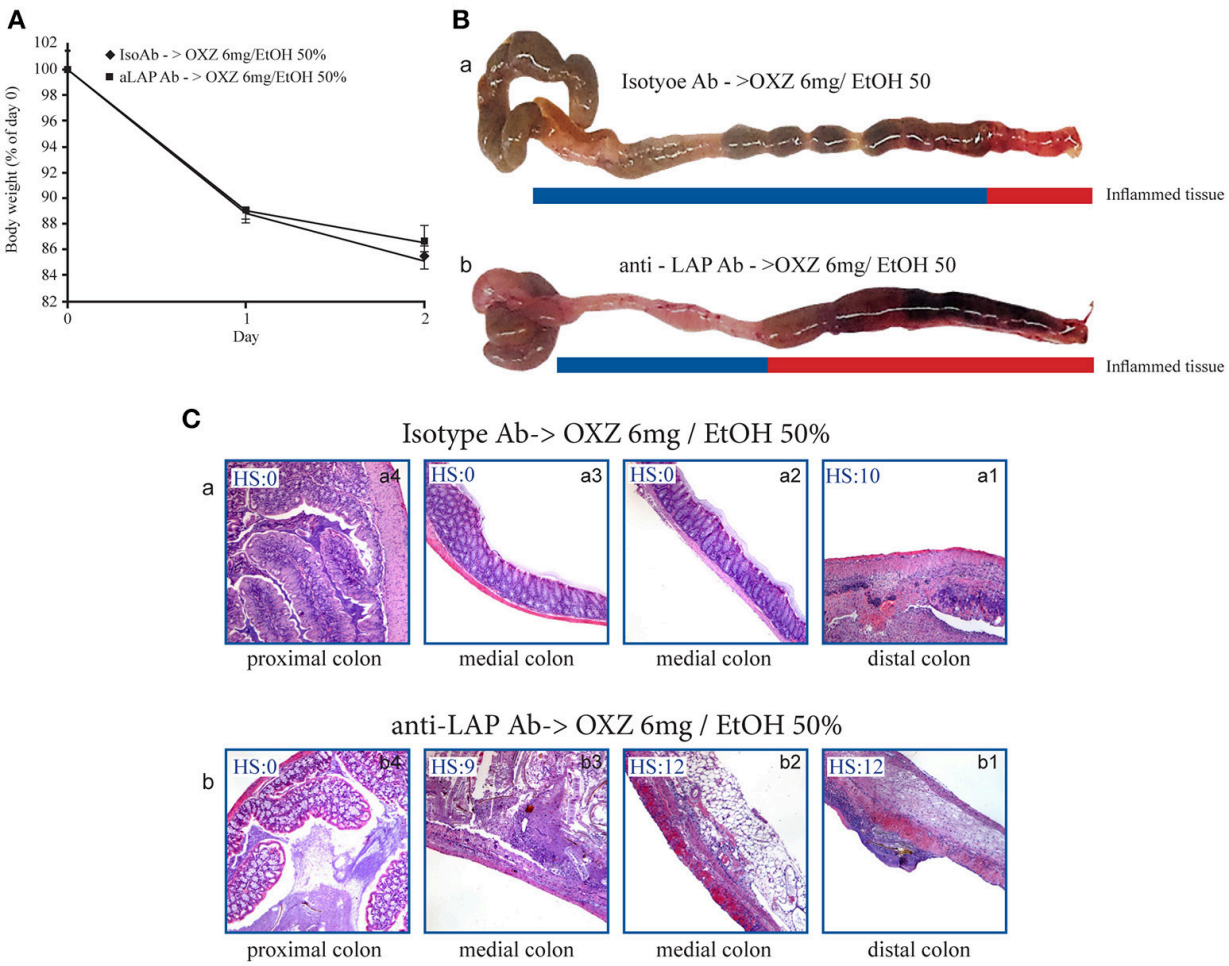
Depletion of LAP+ cells is associated with proximal extension of inflammatory lesions in the mouse oxazolone-induced colitis model. **(A)** Weight changes in oxazolone-treated mice (6 mg/ethanol 50%) pretreated with either Isotype Ab or anti-LAP Ab. Each point represents the cumulative mean (± SE) weight from 3 separate experiments in which 5 mice per group were studied. **(B)** Representative macroscopic images of colons of oxazolone-treated mice pretreated with (a) Isotype Ab or (b) anti-LAP Ab. **(C)** H&E stained colonic specimens (40 × magnification) of distal, medial and proximal colonic portions of oxazolone-treated mice pretreated with (a) Isotype Ab (a1-a4) or (b) anti-LAP Ab Isotype Ab (b1-b4). HS, histopathologic score (see methods).

To gain further insight into the roles of the two aforementioned Tregs subsets in limiting the extension of inflammatory lesions, we evaluated the effect of *in vivo* administration of an LAP-depleting antibody that has no effect on the frequency of CD4+Foxp3+ cells (17) in mice with oxazolone-induced experimental colitis (16). Intrarectal administration of oxazolone (6 mg in 50% ethanol) was associated with weight loss and the onset of distal colitis (Figures 4A,B), as previously described (16). This colitis was associated with significantly higher percentages of LP CD3+CD4+LAP+ cells (Figure 4C) in uninvolved tissue as compared with both involved tissue and with tissue from untreated control mice, and a higher percentage of CD3+CD4+Foxp3+ cells in involved tissue as compared with both uninvolved tissue and control untreated mice (Figure 4D), thus reproducing the observations made in UC patients. In oxazolone colitis, the significant increased % of CD4+LAP+ cells in uninvolved vs. involved tissue was confirmed also by confocal microscopy (Figures 4E,F). After a preliminary validation of the ability of anti-LAP antibody administration to selectively deplete LP CD4+LAP+cell without affecting the % of LP CD4+Foxp3+ cells (Supplementary Figure 2), we administered the antibody or its isotype control in two groups of mice, before the induction of oxazolone colitis. Administration of anti-LAP antibody had no effect on weight loss, which was comparable to that observed in isotype-treated mice (Figure 5A), but was associated with more extensive colitis (Figures 5B,C and Table 2).

**Table 2:** Histopathologic score of colons of mice with oxazolone colitis administered the anti-LAP antibody or its isotype control.

**Treatment**	**Mouse**								**Total**
			A	B	C	D	E	F	(A+B+C+D+E+F)
			0–3	0–3	0–3	0–3	0–3	0–3	0–15
aLAPAb->OXZ 6mg	A	distal colonic tract	0	2	0	3	2	2	9
		medial colonic tract	0	2	0	3	2	0	7
		proximal colonic tract	0	0	0	0	0	0	0
aLAPAb->OXZ 6 mg	B	distal colonic tract	0	2	0	2	2	1	7
		medial colonic tract	0	1	0	2	1	1	5
		proximal colonic tract	0	0	0	0	0	0	0
aLAPAb->OXZ 6 mg	C	distal colonic tract	0	3	0	3	3	3	12
		medial colonic tract	0	2	0	2	3	2	9
		proximal colonic tract	0	0	0	0	0	0	0
IsoAb->OXZ 6 mg	D	distal colonic tract	0	3	0	3	3	3	12
		medial colonic tract	0	0	0	0	0	0	0
		proximal colonic tract	0	0	0	0	0	0	0
IsoAb->OXZ 6 mg	E	distal colonic tract	0	3	0	3	2	2	10
		medial colonic tract	0	0	0	0	0	0	0
		proximal colonic tract	0	0	0	0	0	0	0
IsoAb->OXZ 6 mg	F	distal colonic tract	0	2	0	3	3	1	9
		medial colonic tract	0	0	0	0	0	0	0
		proximal colonic tract	0	0	0	0	0	0	0
								**Mean**	**SE**
aLAPAb->OXZ 6 mg		distal colonic tract						9	1.8
		medial colonic tract						7	1.4
		proximal colonic tract						0	0.0
IsoAb->OXZ 6 mg		distal colonic tract						10	1.1
		medial colonic tract						0	0.0
		proximal colonic tract						0	0.0

*Colons were divided into three sections (proximal, medial, and distal), multiple serial sections were done and slides were scored according with 5 criteria from 0 to 3 (A, hypervascularization; B, presence of mononuclear cells; C, epithelial hyperplasia; D, epithelial damage; E, presence of granulocytes; F, mucosal hemorrhages) to produce a cumulative histopathologic score (HS) ranging from 0 (no colitis) to 15 (maximal colitis activity)*.

## Discussion

The present findings suggest that LP CD3+CD4+LAP+ Tregs are responsible for limiting the extension of inflammatory lesions in UC. We observed increases in both the percentage of CD3+CD4+LAP+ cells and the proportion of IL-10-expressing CD3+CD4+LAP+ cells associated with a reduction of IL-17-expressing CD3+CD4+LAP+ cells in uninvolved vs. involved tissue from UC patients. Previous studies have reported increases in the numbers of CD3+CD4+LAP+ cells (14) and CD3+CD4+Foxp3+ cells (24, 25) in involved UC tissue, the former were found to be functionally impaired *in vitro*, while the latter, although functional *in vitro*, were ineffective in counteracting inflammation *in vivo*. Indeed, in the present study, uninvolved tissue is characterized by a significant increase in the ratio between IL-10 and IL-17 expressing LAP+ cells suggesting that the increased % of CD3+CD4+LAP+ cells observed in uninvolved tissue is predominantly represented by functional active regulatory cells. This hypothesis is reinforced by the observations obtained in mice since selective depletion of LAP+ cells in a mouse model of distal colitis was associated with the extension of inflammatory lesions to the proximal colon. Taken together, these data strongly suggest that CD3+CD4+LAP+ regulatory cells play a key role in limiting the extension of colonic inflammatory lesions in UC. CD3+CD4+LAP+ cells (which express surface TGF-β linked to its latency associated peptide [LAP]) have been shown to limit inflammation in the adoptive transfer mouse model of experimental colitis (28) and are crucial to ensure protection against murine TNBS colitis, since transfer of CD4+LAP+ depleted cells with intact CD4+Foxp3+ cells does not prevent TNBS colitis in recipient mice (29). In mice, the frequency of CD3+CD4+LAP+ cells is increased in conditions of increased intestinal permeability and during the homeostatic response to transient increases in intestinal permeability (29, 30). Furthermore, the frequency of these cells in the intestinal LP is increased in human patients with UC but not Crohn's disease (14), and their regulatory activity is significantly reduced in inflamed UC tissue due to an increase in the proportion of IL-17-expressing LAP+ cells (14). Studies in animal models of autoimmune diseases have shown that intracellular expression of IL-10 is linked to the ability of CD4+LAP+ cells to attenuate disease severity (26, 27). In the present study, we found that the proportion of CD3+CD8- (CD4) LAP+ cells expressing intracellular IL-10 was significantly higher in uninvolved vs. involved tissue from UC patients, suggesting a regulatory role of these cells, which may limit the extension of inflammatory lesions. Nasal administration of anti-CD3 in animal models induces suppressive IL-10-producing T cells (Tr1 cells), almost 75% of which exhibit cell surface expression of LAP (31). The link between the Tr1 cell subset and the CD3+CD4+LAP+ Foxp3-cells described in the present study remains unknown, and further studies will be necessary to clarify this relationship. However, our findings suggest that the suppressive capabilities of these cells are dependent on LAP expression, since depletion of LAP+ cells in mice with oxazolone-induced colitis was associated with proximal extension of colonic inflammatory lesions. Supporting this view, we previously demonstrated that blockade of TGF-β activity in oxazolone-induced colitis is associated with the development of extensive colitis (16).

We found that while the proportion of CD3+CD4+LAP+ cells was increased in uninvolved tissue, the proportion of CD3+CD4+Foxp3+ cells in the same tissue was comparable to that seen in controls, suggesting that the requirements for CD3+CD4+LAP+ cell expansion differ to those for CD3+CD4+Foxp3+ cell expansion. In mice, *in vivo* expansion of CD3+CD4+LAP+ cells is dependent on the presence of IL-10 and TLR2 (21, 29, 32), and the presence of TGF-β, acting in an autocrine fashion, is required for LAP expression independently of Foxp3 expression (22). It is therefore tempting to speculate that in uninvolved tissue, TGF-β levels facilitate the maintenance and expansion of CD3+CD4+LAP+ regulatory cells but are insufficient to induce CD3+CD4+Foxp3+ Tregs.

It is increasingly apparent that microbiota play an important role in modulating the intestinal immune system, influencing different immune-cell subsets and their soluble products. We previously reported that the expansion of CD3+CD4+LAP+ cells in mice is dependent on the presence of microbiota (29, 30), and showed that probiotic administration increases the percentage of LP CD3+CD4+LAP+ cells and protects mice from TNBS-induced colitis (32). Moreover, we have demonstrated that probiotic administration in patients with ileal pouch anal anastomosis for ulcerative colitis is associated with an increased percentage of CD3+CD4+LAP+ cells in the pouch LP and a decrease in pouch disease activity index (13). Taken together, these observations suggest that expansion of LP CD3+CD4+LAP+ cells may constitute a therapeutic strategy to limit and possibly prevent colonic inflammation in UC patients. Microbiota modulation might represent a useful tool to accomplish this task.

## Author contributions

Study conception and design: MB and RP. Study supervision: MB. Acquisition of data: AB, AA. Immunofluorescence and experimental colitis: ND. Experimental colitis: NC. Flow cytometry analysis supervision: MS. Patients' enrollment, endoscopy, and biopsies collection: AP and RP. Clinical data collection: DD. Histology and histological score: FB. Confocal microscopy: RL and LF. Analysis and interpretation of data: AB, MS, FB, MB, AP, RP, RL, and LF. Drafting of manuscript: MB. Critical revision: AB, AP, RP, and MB. The final version of the manuscript has been approved by all authors.

### Conflict of interest statement

The authors declare that the research was conducted in the absence of any commercial or financial relationships that could be construed as a potential conflict of interest.
