# Supplementary figures and images for: CD3+CD4+LAP+Foxp3-Regulatory Cells of the Colonic Lamina Propria Limit Disease Extension in Ulcerative Colitis
Source: Front Immunol. 2018 Oct 30;9:2511. doi: 10.3389/fimmu.2018.02511 (PMC6219428; doi:10.3389/fimmu.2018.02511)

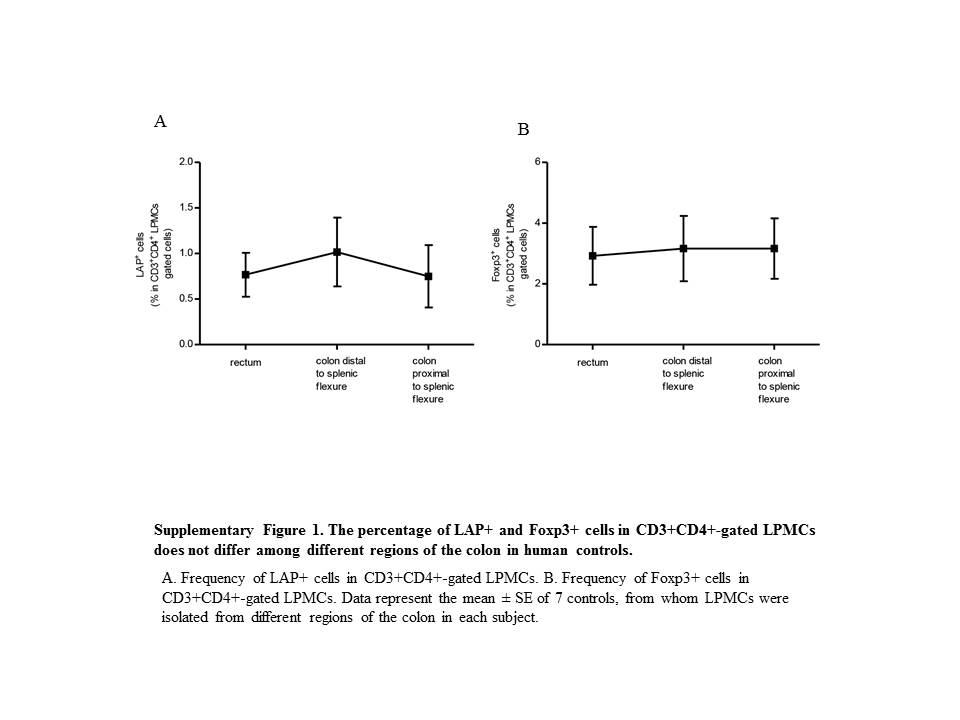

Supplement: Supplementary file 1 [file Image_1.JPEG]

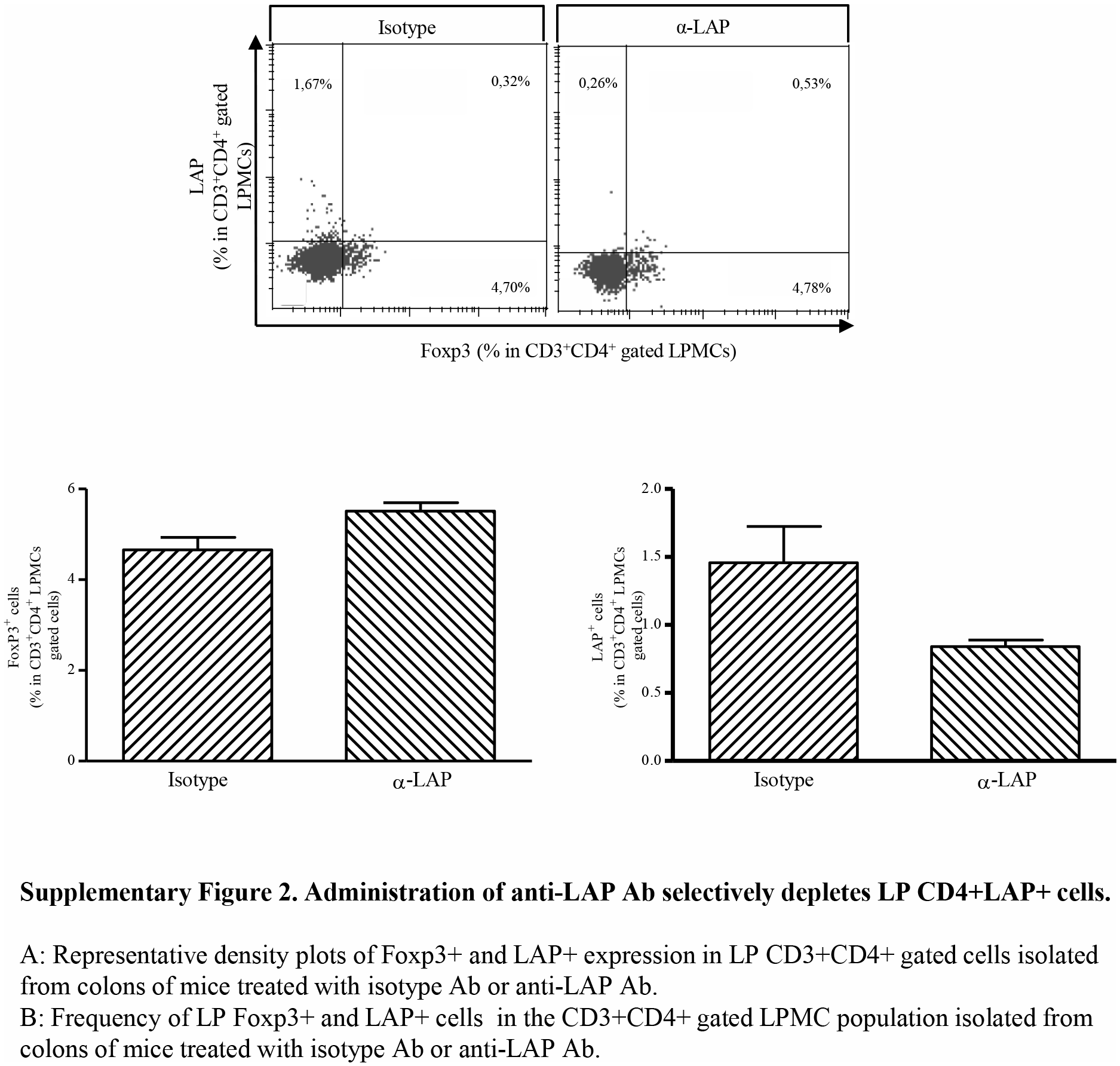

Supplement: Supplementary file 2 [file Image_2.tif]
